# Decreased connection density and modularity of functional brain networks during n‐back working memory paradigm

**DOI:** 10.1002/brb3.1932

**Published:** 2020-11-13

**Authors:** Zalan Kaposzta, Orestis Stylianou, Peter Mukli, Andras Eke, Frigyes Samuel Racz

**Affiliations:** ^1^ Department of Physiology Semmelweis University Budapest Hungary

**Keywords:** brain, cognition, electroencephalography, functional connectivity, working memory

## Abstract

**Introduction:**

Investigating how the brain adapts to increased mental workload through large‐scale functional reorganization appears as an important research question. Functional connectivity (FC) aims at capturing how disparate regions of the brain dynamically interact, while graph theory provides tools for the topological characterization of the reconstructed functional networks. Although numerous studies investigated how FC is altered in response to increased working memory (WM) demand, current results are still contradictory as few studies confirmed the robustness of these findings in a low‐density setting.

**Methods:**

In this study, we utilized the n‐back WM paradigm, in which subjects were presented stimuli (single digits) sequentially, and their task was to decide for each given stimulus if it matched the one presented n‐times earlier. Electroencephalography recordings were performed under a control (0‐back) and two task conditions of varying difficulty (2‐ and 3‐back). We captured the characteristic connectivity patterns for each difficulty level by performing FC analysis and described the reconstructed functional networks with various graph theoretical measures.

**Results:**

We found a substantial decrease in FC when transitioning from the 0‐ to the 2‐ or 3‐back conditions, however, no differences relating to task difficulty were identified. The observed changes in brain network topology could be attributed to the dissociation of two (frontal and occipitotemporal) functional modules that were only present during the control condition. Furthermore, behavioral and performance measures showed both positive and negative correlations to connectivity indices, although only in the higher frequency bands.

**Conclusion:**

The marked decrease in FC may be due to temporarily abandoned connections that are redundant or irrelevant in solving the specific task. Our results indicate that FC analysis is a robust tool for investigating the response of the brain to increased cognitive workload.

## Graphical abstract

In this study, we investigated functional brain connectivity of young healthy volunteers while performing an n‐back working memory paradigm. Our results indicated a marked decrease in connectivity strength and network modularity when transitioning from control (0‐back) to test conditions (2‐ and 3‐back), while no difference associated with task difficulty could be identified. These results reflected the dissolution of two functional modules (a frontal and an occipitotemporal) that were only present in the control condition.

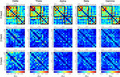

## 1 INTRODUCTION

The brain is a complex system of small functional units—neurons—that are densely interconnected and thus functionally coupled through thousands of synapses per neuron (Sporns et al., 2005). This structural and functional organization lays the foundation for emergent behavior, that is, the ability of the brain to perform substantially more complex tasks than it could be predicted based solely on the functional capacities of its constitutive elements (Chialvo, 2010; Werner, 2010). Emergence is considered to be achieved through quick formation and dissolution of functionally coupled neuronal assemblies (Friston, 2000a, 2000b), ranging in size from neural microcircuits through macroanatomical cortical regions devoted to specific functions to whole‐brain functional networks (Bullmore et al., 2009; Bullmore & Sporns, 2009). Although the mechanisms of coordinated activity concerning disparate regions of the brain has been the subject of intense research for almost three decades now (Biswal et al., 1995; Friston et al., 1993), it is still poorly understood how functional reorganization on the whole‐brain level is related to increased mental workload, especially when various difficulty levels at the same task are applied.

The analysis of functional connectivity (FC) has been proven to be a robust and efficient tool for capturing the large‐scale functional organization of the brain (Sporns, 2011; van den Heuvel & Hulshoff Pol, 2010). Numerous methods have been proposed to characterize FC, with the graph theory‐based approach being one of the most popular given its relatively simple interpretability and strong expressive power (Bullmore & Sporns, 2009). Within a graph theoretical FC framework, the brain is modeled as a network whose nodes are the investigated brain regions while its edges represent the functional coupling between the corresponding regions. Nodes of the network can be defined based on a priori hypotheses—for example Brodmann areas—or are inherently determined by the utilized imaging technique itself, for example, by the recording sites of an electroencephalography (EEG) system. The functional cooperation between the nodes could then be estimated from regional activities with a plethora of statistical methods, each with its own advantages and disadvantages (Bastos & Schoffelen, 2016). Finally, the obtained networks can be quantitatively described by a set of graph theoretical measures, capable of characterizing various aspects of network topology (Rubinov & Sporns, 2010). This relatively simple concept was indeed demonstrated to be a powerful asset of neuroscience, which could be utilized for a better understanding of not only physiological but also pathological brain function (Bullmore & Sporns, 2009; Stam, 2014). Furthermore, the graph theoretical FC approach was successfully applied for characterizing the effects of various cognitive stimulation paradigms as well (Hou et al., 2018; Racz et al., 2017; Ren et al., 2017).

Working memory (WM) paradigms provide means for investigating how brain networks reconfigure themselves in response to increased mental workload. In a WM paradigm, transient storage and processing of the information is a prerequisite to solve a complex cognitive task (Baddeley, 2003). Moreover, WM paradigms elicit coordinated responses in multiple brain areas (Cohen & D'Esposito, 2016), with the prefrontal cortex (PFC) often playing a central role (Aghajani et al., 2017; Cohen et al., 1997). The *n*‐back paradigm (Kirchner, 1958) is among the most frequently utilized WM tasks (Owen et al., 2005), in which the level of difficulty can be adjusted such that a state of increased mental workload can be maintained in the range of tens of seconds. This is key in obtaining statistically reliable connectivity estimates that are free of transients. Assessing FC in subjects while performing *n*‐back tasks could not only enhance our understanding of how the brain adapts to various levels of cognitive challenges but would also allow for disentangling what characteristics of large‐scale neural networks are related to cognitive or behavioral performance.

Although multiple studies investigated FC during an *n*‐back WM task, results are somewhat contradictory. On one hand, findings in EEG studies are consistent in that functional segregation and connectivity strength decreases during tasks when compared to rest (Cohen & D'Esposito, 2016; Ginestet & Simmons, 2011; Hou et al., 2018). On the other hand, functional integration has been shown to decrease (Hou et al., 2018) and increase (Cohen & D'Esposito, 2016; Dai et al., 2017) alike while performing an *n*‐back task. Furthermore, measures of global FC were found increased during *n*‐back stimulation (Fishburn et al., 2014) when monitoring neural activity with functional near‐infrared spectroscopy (fNIRS). Functional magnetic resonance imaging (fMRI) studies reported on both decreased and increased FC for the default mode‐ and dorsal attention networks, respectively (Godwin et al., 2017; Newton et al., 2011). Task difficulty either had no (Hou et al., 2018) or an ambiguous effect on FC (Newton et al., 2011) and only weak correlations were found between FC network characteristics and behavioral measures (Cohen & D'Esposito, 2016; Dai et al., 2017; Hou et al., 2018). Comparing previous results is further complicated by the fact that most studies utilized different imaging techniques, preprocessing pipelines as well as slight variations in implementing the *n*‐back paradigm.

In this study, we set out to investigate the effects of the *n*‐back WM task on whole‐brain FC when cortical activity is assessed with a relatively low‐density EEG setup. This would not only allow us to characterize how large‐scale brain networks are reorganized during graded mental workload but also to evaluate how robust the elicited changes are, which latter aspect of our study is aimed at broadening the applicability of WM related tests. The observed measures of global FC decreased markedly when transitioning from control to task states, which was found to be most prominent over the prefrontal and frontal regions. On the other hand, graded levels of task difficulty could not be distinguished based on their connectivity characteristics. Weak correlations between behavioral and FC measures were also revealed, mostly over prefrontal regions.

## 2 MATERIALS AND METHODS

### 2.1 Participants

For this study, 56 young, healthy volunteers were recruited (mean age of 24.3 years, 30 females). The study was designed according to the standards of the Declaration of Helsinki, it was approved by the Semmelweis University Regional and Institutional Committee of Science and Research Ethics (approval number: 2017/94), and all participants provided written informed consent prior to the measurement. Only individuals over the age of 18 were allowed to participate. Subjects were instructed not to consume any substances affecting cognitive performance (e.g., caffeine) for at least 3 hr prior to measurement, and to have at least 6 hr of sleep the preceding night. Exclusion criteria included neuropsychological or psychiatric morbidities, brain damage, ongoing medication affecting the central nervous system, pregnancy, or the presence of any general medical condition. All participants were able to perform the measurement protocol; however, five female and three male subjects were later excluded from further analysis due to poor signal quality or excessive head motion. Thus, the final sample included 25 female (2 left‐handed, mean age of 24.8 ± 3.16 years) and 23 male (3 left‐handed, mean age of 24.0 ± 2.68 years) participants, resulting in a total number of 48 subjects.

### 2.2 The n‐back paradigm and behavioral measures

In the *n*‐back WM paradigm, participants are presented visual stimuli (e.g., Arabic numbers in the range 0–9) in a pseudorandom sequence that they have to recall later from their short‐term memory. Accordingly, for each new stimulus presented the task is to decide if the current item is the same as the one presented *n* items prior (Sweet, 2011). Participants have to provide a response for each and every stimulus, for example, in a 2‐back task (*n* = 2) for every item presented, they had to answer “yes” if it matches the one presented 2 items ago, while “no” otherwise.

**FIGURE 1:**
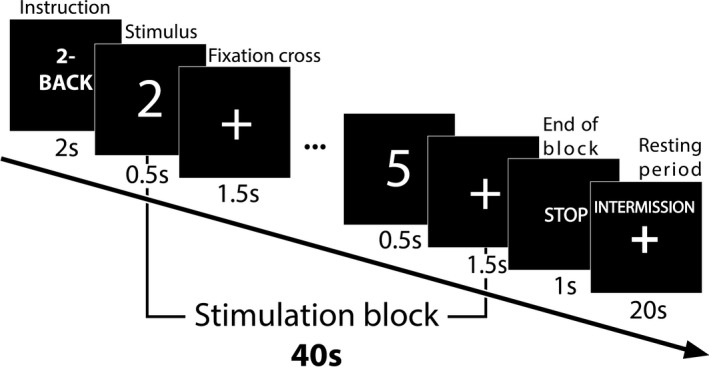
Schematic diagram of the n‐back experimental protocol. The timing sequence of all sessions is illustrated on one session of 2‐back task. First, the difficulty level is shown for 2 s, followed by a stimulation block of 40s. Throughout the stimulation block, digits are presented sequentially at every 2s, with the stimuli being visible for 0.5s and followed by a fixation cross for 1.5s. The end of the stimulation block is indicated by a ‘STOP’ screen presented for 1 s, which is followed by a resting period of 20s showing only the fixation cross

For this study, we adapted the *n*‐back protocol as reported in Shin et al. (2018). In that, participants performed *n*‐back tasks at two difficulty levels (2‐ and 3‐back) while 0‐back tasks were carried out for baseline acquisition. The presented stimuli were single‐digit Arabic numbers ranging from 0 to 9. Following a brief practice session ensuring that the task was understood, each subject had to complete three measurement sessions with one session containing three 0‐, 2‐ and 3‐back stimulus blocks, yielding 9 stimulus blocks for each difficulty level and 27 stimulus blocks in total. Within each session, the three task types were arranged in a counterbalanced order so to provide variability in the difficulty level. The schematic diagram of a stimulation block is shown in Figure 1. Each task started with a 2‐s instruction screen showing the type of the upcoming task. This was followed by a 40‐s stimulus block comprising of 20 sequential stimuli. For every trial, the stimulus symbol was visible for 0.5 s and was subsequently replaced by a fixation cross for 1.5 s. The stimulus block was then followed by a 1‐s long “stop” screen and a 20‐s long resting period before the next instruction screen. Each stimulation block contained a “target” number that had an increased chance of appearing, creating 6 positive and 14 negative trials in a randomized order (Shin et al., 2018). During the 2‐ and 3‐back tasks, participants had to press the “yes” button (number 7 on keyboard) with their right index finger in case of positive stimuli (i.e., if the presented number matched the one showed 2‐ or 3‐times earlier, respectively), while the “no” key (number 8 on keyboard) with their right middle finger otherwise. During the 0‐back paradigm, participants were instructed to press the key assigned to a “yes” response for each stimulus in order to maintain subject attention. A one‐minute resting period was included between the measurement sessions where subjects were allowed to move or speak if necessary. The n‐back protocol was implemented in Matlab (MathWorks).

For each stimulus, subject reaction time (RT) was assessed and registered as the time from stimulus onset to the response. If the subject was unable to provide a response during a trial, RT was automatically set to 2 s. Reaction times within each 40‐s stimulation block were averaged. Finally, the effective RT for the 0‐, 2‐ and 3‐back tasks was obtained as the grand average taken over the 9 stimulation blocks for the corresponding difficulty levels. Cognitive performance was assessed for each stimulation block through response accuracy (ACC), defined as the fraction of correct (including both positive and negative) responses given for the 20 trials presented in the block. The effective ACC for the three difficulty levels again was obtained by averaging accuracy values over the nine corresponding stimulation blocks.

### 2.3 Data acquisition

Measurements were implemented in a dark, electrically sealed room, where participants were seated in a comfortable armchair in front of a computer display and a keyboard. EEG data acquisition was carried out by using an Emotiv Epoc + wireless EEG system and the EmotivPRO software (Emotiv Systems Inc., San Francisco, CA, USA). The device allowed the monitoring of neural activity from 14 standard locations according to the international 10‐10 system (AF3, AF4, F3, F4, F7, F8, FC5, FC6, T7, T8, P7, P8, O1, and O2) with reference (CMS) and ground (DRL) electrodes positioned at the left and right mastoid processes. EEG was recorded at 2048 Hz and was internally downsampled to 128 Hz. The data was internally band‐pass filtered between 0.2 and 48 Hz using a 5th order digital Sinc filter as well as additional notch filters were applied at 50 and 60 Hz. Electrode impedances were kept under 20 kΩ so that all measurements could be carried out with maximal contact quality, as indicated by the EmotivPRO software.

### 2.4 Data preprocessing

Raw EEG data were first segmented into 40‐s epochs, containing only the stimulation periods. Epochs were band‐pass filtered using a 5th order zero‐phase Butterworth filter with lower and upper cutoff frequencies of 0.5 and 45 Hz. Artifact removal was performed for every epoch separately using the EEGLAB toolbox (Delorme & Makeig, 2004). In that, independent component analysis (ICA) was used to decompose the data (Hyvarinen & Oja, 2000) into 14 linearly maximally independent components. From these components, those that could be associated with eye movement, blinking, muscle contractions, or cardiac activity were identified and excluded before performing reverse ICA. Subsequently, epochs were visually inspected, and artifact‐free segments of 36 s were selected for further analysis. Finally, data were again band‐pass filtered using a 5th order zero‐phase Butterworth filter for five frequency bands traditionally used in EEG analysis: delta (0.5–4 Hz), theta (4–8 Hz), alpha (8–13 Hz), beta (13–30 Hz), and gamma (30–45 Hz).

### 2.5 Functional connectivity estimation

The phase lag index (PLI, Stam et al., 2007)) was used to estimate FC between all pairs of brain regions. PLI is a measure of phase synchronization (Rosenblum et al., 1996), according to which two processes are considered phase‐locked if the difference between their instantaneous phases (Δφ) is constant or at least bounded between a small interval. The latter—that represents a weaker concept of phase coupling—can be expressed as

(1) $$\left|\Delta \varphi \right|=\left|\varphi_{1}‐\varphi_{2}\right|<c,$$

where $\varphi_{1}$ and $\varphi_{2}$ are the phases of the two processes acquired by Hilbert transform and *c* is a constant less than 2π. In EEG‐based connectivity studies however, due to volume conduction or active reference electrodes, this measure is susceptible to the effects of common sources, which may result in spuriously high connectivity estimates between channel pairs (Stam et al., 2007). PLI accounts for this by discarding phase differences centered around 0 mod π indicating instantaneous coupling, that is more likely to originate from common source effects. This is achieved by looking at the distribution of phase differences, as an asymmetric distribution would indicate a stable phase‐locking with a non‐zero Δφ. Therefore, an asymmetry index can be obtained from the time series of phase differences ($\Delta \varphi \left(t\right)=\varphi_{1}\left(t\right)‐\varphi_{2}\left(t\right)$), where $\varphi_{1}\left(t\right)$ and $\varphi_{2}\left(t\right)$ are the instantaneous phases of the two processes), that yields the definition of PLI (Stam et al., 2007):

(2) $$\text{PLI}=\left|\left\langle sign[\Delta \varphi (t)]\right\rangle \right|,$$

where ⟨·⟩ denotes the mean and $\left|\cdot \right|$ denotes the absolute value function. PLI is bound between 0 and 1 with 1 indicating perfect phase‐locking (with Δφ≠0modπ), 0 indicating uncoupled dynamics and larger PLI values implying stronger functional coupling (Stam et al., 2007).

In every stimulation block, PLI was calculated for all channel pairs, yielding a 14 × 14 weighted connectivity matrix. For each difficulty level (0‐, 2‐ and 3‐back), the nine matrices obtained from the stimulation blocks were averaged in order to increase the signal‐to‐noise ratio and capture the characteristic connectivity patterns of the various mental workload levels, and the average matrices were used subsequently for calculating graph theoretical measures. This technique was shown to provide more reproducible connectivity estimates than calculating network measures first and averaging them afterward (Hardmeier et al., 2014). No additional thresholding was applied to the averaged connectivity matrices that were then made subject to graph theoretical analysis.

### 2.6 Graph theoretical analysis

Each averaged connectivity matrix defined a weighted, undirected, fully connected network: Nodes of the network representing the monitored brain regions, edges of the network representing functional connections between corresponding regions, and edge weights—given by the average PLI values—indicating the strength of coupling between the corresponding regions. These networks can be characterized by a set of graph theoretical metrics, each providing insight on different aspects of network structure and function (Bullmore & Sporns, 2009; Rubinov & Sporns, 2010). Considering the small size of the reconstructed networks, the following set of global and local network metrics were calculated in this study using functions of the Brain Connectivity Toolbox (Rubinov & Sporns, 2010).

#### 2.6.1 Connection density and weighted node degree

For each node *d* of the network, the weighted node degree (*D_w_*) was obtained by summing the weights of edges connected to *d*. Global connection density ($\bar{D_{w}}$) was acquired as averaging *D_w_* within the network. Connection density is often considered to reflect the cost of functional networks, while weighted node degree characterizes the connectedness of node *d* to the rest of the network (Rubinov & Sporns, 2010).

#### 2.6.2 Clustering coefficient

The clustering coefficient of a particular node (*C_w_*) can be defined as the fraction of the node's neighbors that are also neighbors of each other (Watts & Strogatz, 1998). Although this original concept applies for binary networks (where edge weights are either 0 or 1 depending on the existence of a link between the corresponding nodes), the concept of clustering can be extended to weighted networks as well (Onnela et al., 2005), yielding *C_w_*. The global clustering coefficient ($\bar{C_{w}}$) is the average of *C_w_* taken over all nodes of the network. $\bar{C_{w}}$ and *C_w_* characterize network segregation, that is, the presence of smaller node groups (clusters) within the network that are densely interconnected with each other.

#### 2.6.3 Global and local efficiency

Global efficiency of a network is the average inverse shortest path length between two randomly selected nodes (Latora & Marchiori, 2001). The weighted global efficiency ($\bar{E_{w}}$) can be similarly defined for weighted networks by first transforming edge weights into proximity indices, that is, by taking their inverse (Rubinov & Sporns, 2010). It is strongly related to the characteristic path length (the average shortest path length between two randomly selected nodes), however $\bar{E_{w}}$ is often considered as a superior measure, especially in the case of smaller networks (Achard & Bullmore, 2007). Local efficiency (*E_w_*) of node *d* is the efficiency of the subnetwork defined by the neighbors of *d*. $\bar{E_{w}}$ is a measure of network integration, as it reflects how fast information from disparate regions of the network can be combined on the global level. On the other hand, *E_w_* captures very similar information to that of *C_w_*, therefore it is more often considered as a measure of local segregation.

#### 2.6.4 Modularity

The modularity index (*Q*) captures the degree to which the network can be subdivided into nonoverlapping groups of nodes (modules), where within‐group connectivity is maximized while at the same time between‐group connectivity is minimized (Newman, 2004). *Q* is a more sophisticated measure of network segregation than $\bar{C_{w}}$, in that it not only captures the presence of densely connected subgroups but can also provide an exact community structure and composition of such groups (Newman, 2006; Rubinov & Sporns, 2010). The modularity indices and node partitions were obtained using the algorithm described in (Newman, 2004, 2006) with the modularity resolution parameter set to the default value of one. Group‐characteristic modules were defined according to the most frequently obtained partition (i.e., the mode of the community structures obtained among all subjects for the given state).

### 2.7 Statistical analyses

Since data did not meet at least one of the assumptions of normality, homogeneity, and sphericity, nonparametric statistical methods were used to compare behavioral variables, global and local network measures between the three difficulty levels, as well as to estimate correlations between behavioral and connectivity measures.

The effects of task difficulty on behavioral measures RT and ACC were assessed by Friedman tests at significance level α=0.05. Consistency of the results among subjects was estimated using Kendall's *W* coefficient of concordance. Post hoc pairwise analyses were carried out using paired Wilcoxon signed‐rank tests and *p*‐values were adjusted to control for multiple comparisons using Bonferroni method.

Similarly, global network measures ($\bar{D_{w}}$, $\bar{C_{w}}$, $\bar{E_{w}}$ and *Q*) obtained for the three difficulty levels were compared using Friedman tests while concordance among subjects was estimated using Kendall's *W*. Paired Wilcoxon signed‐rank tests were used for post hoc pairwise comparisons with Bonferroni adjustments.

The main effect of localization on local network measures (*D_w_*, *C_w_* and *E_w_*) was assessed by Friedman tests at level α=0.05 for each difficulty level separately and the consistency of regional differences among subjects was estimated using Kendall's *W*. Then, the effect of task difficulty on local network measures was assessed for each channel individually using Friedman tests and Kendall's *W*. Post hoc comparisons were again carried out using paired Wilcoxon signed‐rank tests and *p*‐values were Bonferroni adjusted to account for multiple comparisons.

The plausible relationships between behavioral measures RT and ACC and both global and local network measures were estimated by Spearman rank correlation (*r*) in 0‐, 2‐ and 3‐back conditions separately. Considering the exploratory nature of these results, the *p*‐values obtained for the correlations were not adjusted for multiple comparisons.

## 3 RESULTS

### 3.1 Behavioral measures

**FIGURE 2:**
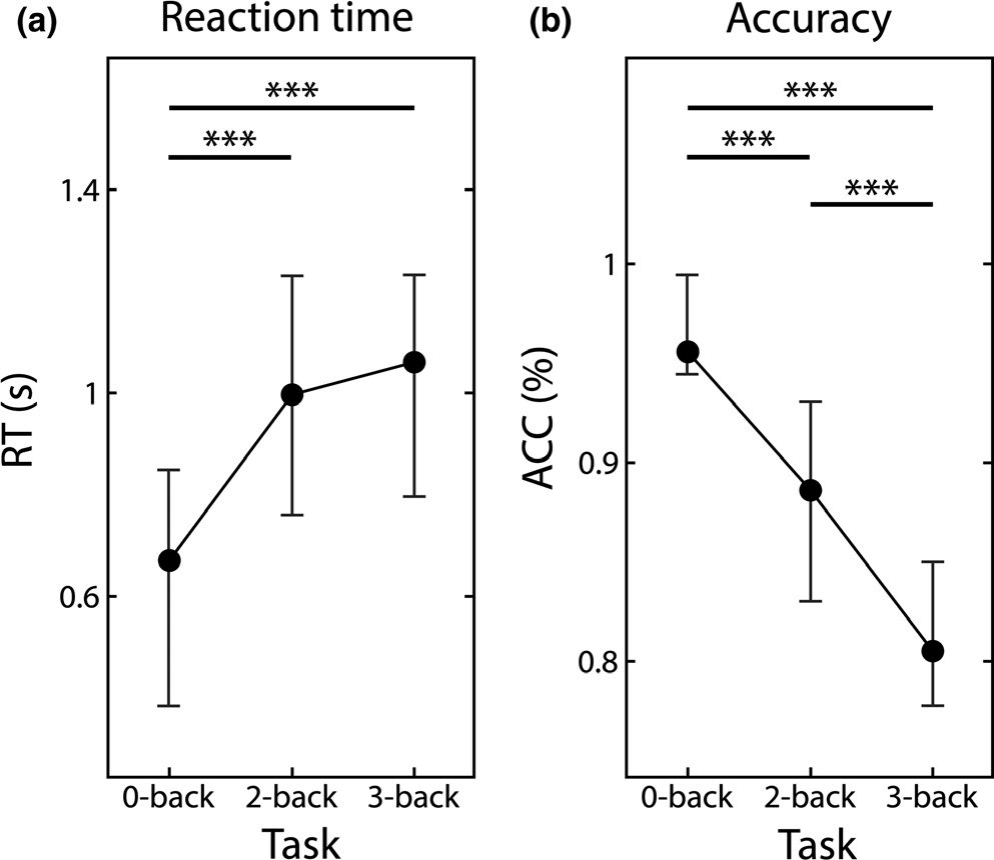
Behavioral variables. Reaction times (a) and accuracy (b) are shown for the three different conditions. Reaction times were found increased in the 2‐ and 3‐back conditions when compared to 0‐back, while accuracy decreased steadily with the increase of task difficulty. Solid circles mark the median values while lover and upper vertical bars denote the 25th and 75th percentiles, respectively. Significant pairwise differences are marked by *** indicating*p* < 10^–6^following Bonferroni adjustment

Task (2‐ and 3‐back) states could be characterized with longer RT when compared to the control (0‐back) state, however, no difference of RT was found between the two difficulty levels (see Figure 2a). As expected, subject accuracy decreased steadily with increasing task difficulty (see Figure 2b).

### 3.2 Global connectivity measures

We found a markedly strong correspondence among subjects between $\bar{D_{w}}$, $\bar{C_{w}}$ and $\bar{E_{w}}$ in all conditions and frequency bands (pairwise Spearman correlation coefficient *r* > .98, *p* < 10^–6^ in all cases), indicating that the three network measures generally captured the same information. Therefore, in order to avoid redundancy, in the followings, we only present results regarding $\bar{D_{w}}$ (as results acquired for $\bar{C_{w}}$ and $\bar{E_{w}}$ were nearly identical) and *Q*.

**FIGURE 3:**
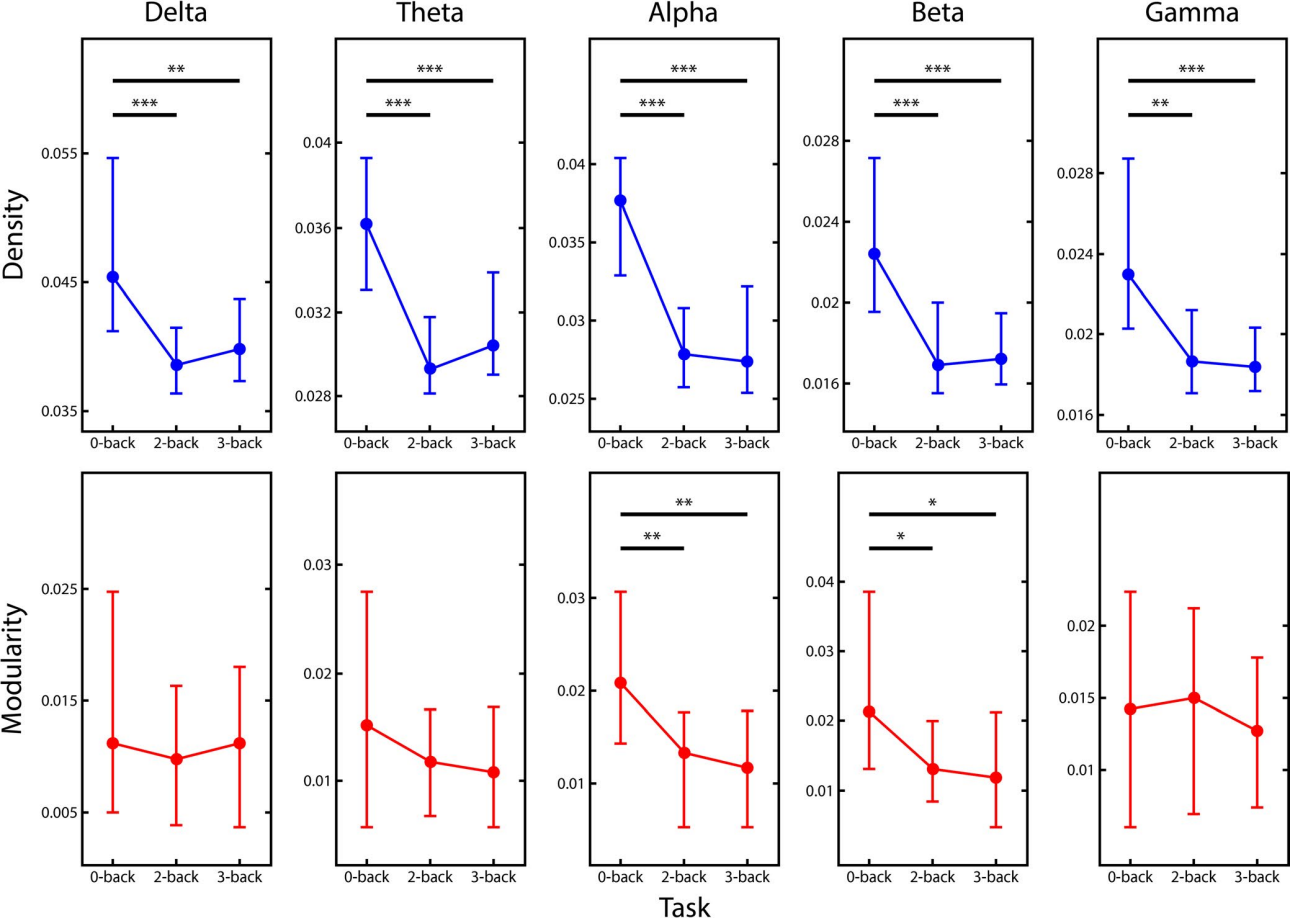
Global network measures. Density (upper row) and modularity (lower row) median values are shown for the three conditions in all five frequency bands (from left to right: delta, theta, alpha, beta and gamma). In all five frequency bands density decreased markedly when transitioning from 0‐ to 2‐ and 3‐back conditions, however no difference was found between the two difficulty levels (2‐ and 3‐back). On the other hand, this same difference in modularity could be observed only in the alpha and beta bands. Significant pairwise differences are marked by *, ** and *** indicating*p* < .05,*p* < 10^–4^and*p* < 10^–6^following Bonferroni adjustment, respectively

In all five frequency bands, connection density reduced significantly during the 2‐ and 3‐back conditions when compared to the 0‐back condition (see Figure 3, upper panel). This pattern was identified as highly consistent among subjects, as indicated by high Kendall's *W* values (Table 1). Pairwise post hoc comparisons indicated robust differences between 0‐back and the two task conditions (*p* < 10^–6^ in nearly all cases, see Figure 3), however, no difference was identified between the two difficulty levels. Modularity also decreased during increased mental workload (see Figure 3, lower panel), albeit this was less pronounced and could be captured significantly only in the alpha and beta bands (Table 1). Although pairwise comparisons indicated a decrease of *Q* during 2‐ and 3‐back in the beta band, the Friedman test failed to identify a significant main effect of state (Table 1). Similarly to $\bar{D_{w}}$, no difference was found between 2‐ and 3‐back conditions.

**TABLE 1:** Friedman test results and Kendall's *W* values for connection density and modularity

Band	Connection density		Modularity	
	Friedman *p*	Kendall's *W*	Friedman *p*	Kendall's *W*
Delta	<10^–6^	0.5135	0.2053	0.0330
Theta	<10^–6^	0.3236	0.0710	0.0551
Alpha	<10^–6^	0.5781	<10^–4^	0.2201
Beta	<10^–6^	0.5473	0.0553	0.0603
Gamma	<10^–6^	0.4236	0.3050	0.0247

**FIGURE 4:**
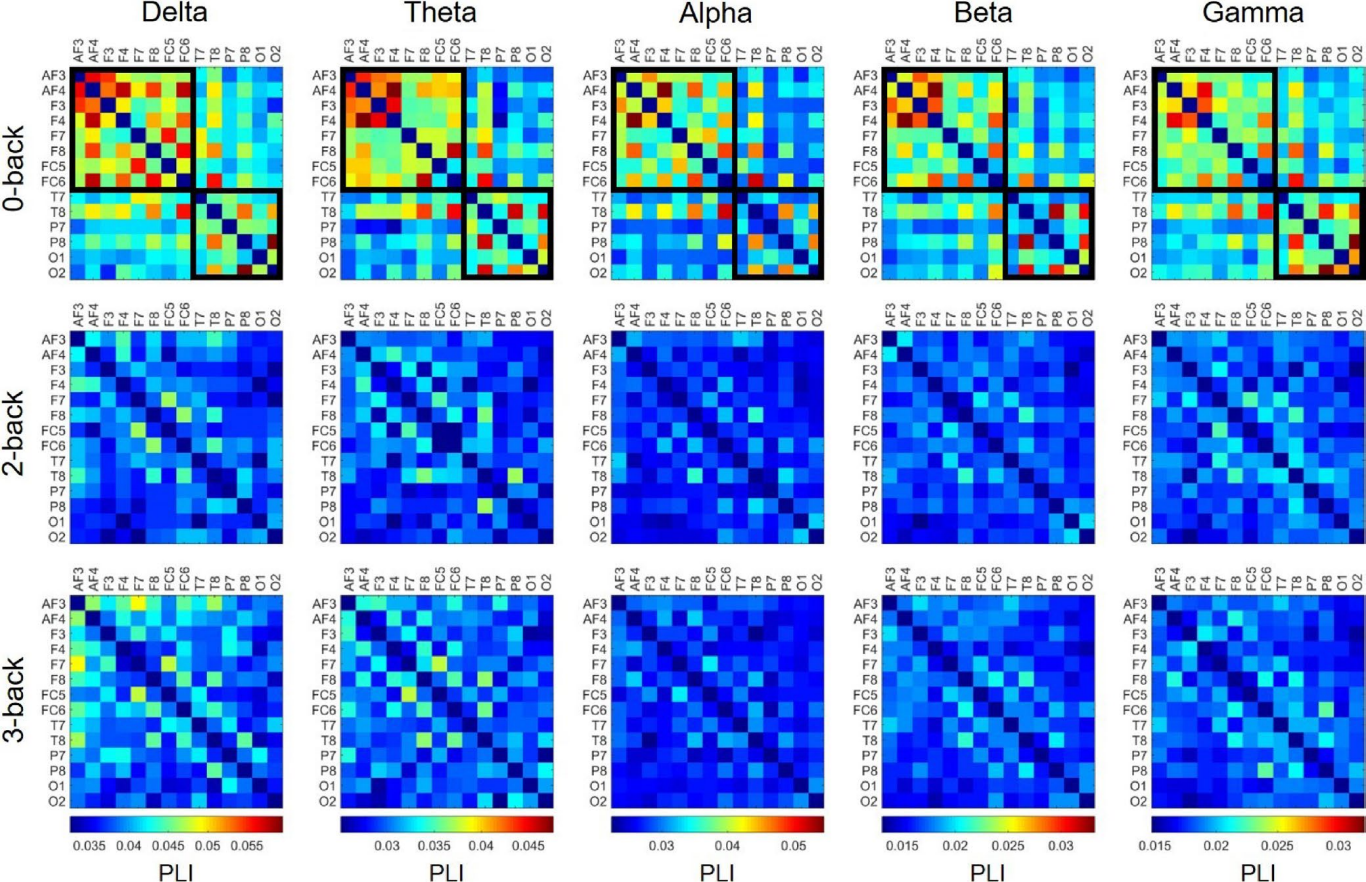
Grand median connectivity matrices. Group‐representative connectivity matrices are shown for every task condition (from top to bottom: 0‐back, 2‐back and 3‐back) in all frequency bands (from left to right: delta, theta, alpha, beta and gamma). Matrices in each frequency band are on the same scale for better comparison. A strong decrease in overall connectivity strength is apparent in all five frequency bands when transitioning from 0‐ to 2‐ and/or 3‐back conditions. The two identified modules (frontal and occipitotemporal) are marked in the 0‐back matrices with black lines. PLI = phase lag index

The above‐described results are also illustrated by the grand median connectivity matrices (i.e., for each cell the median was taken over the corresponding values of the 48 subjects) for the three conditions and five frequency bands (see Figure 4). Reduction in connection density in 2‐ and 3‐back conditions is apparent in all frequency bands. Also, the most frequently identified community structure is illustrated for the 0‐back matrices, indicating the presence of a frontal and an occipitotemporal module, which practically got disassembled in the 2‐ and 3‐back conditions (see Figure 4).

### 3.3 Local connectivity

**FIGURE 5:**
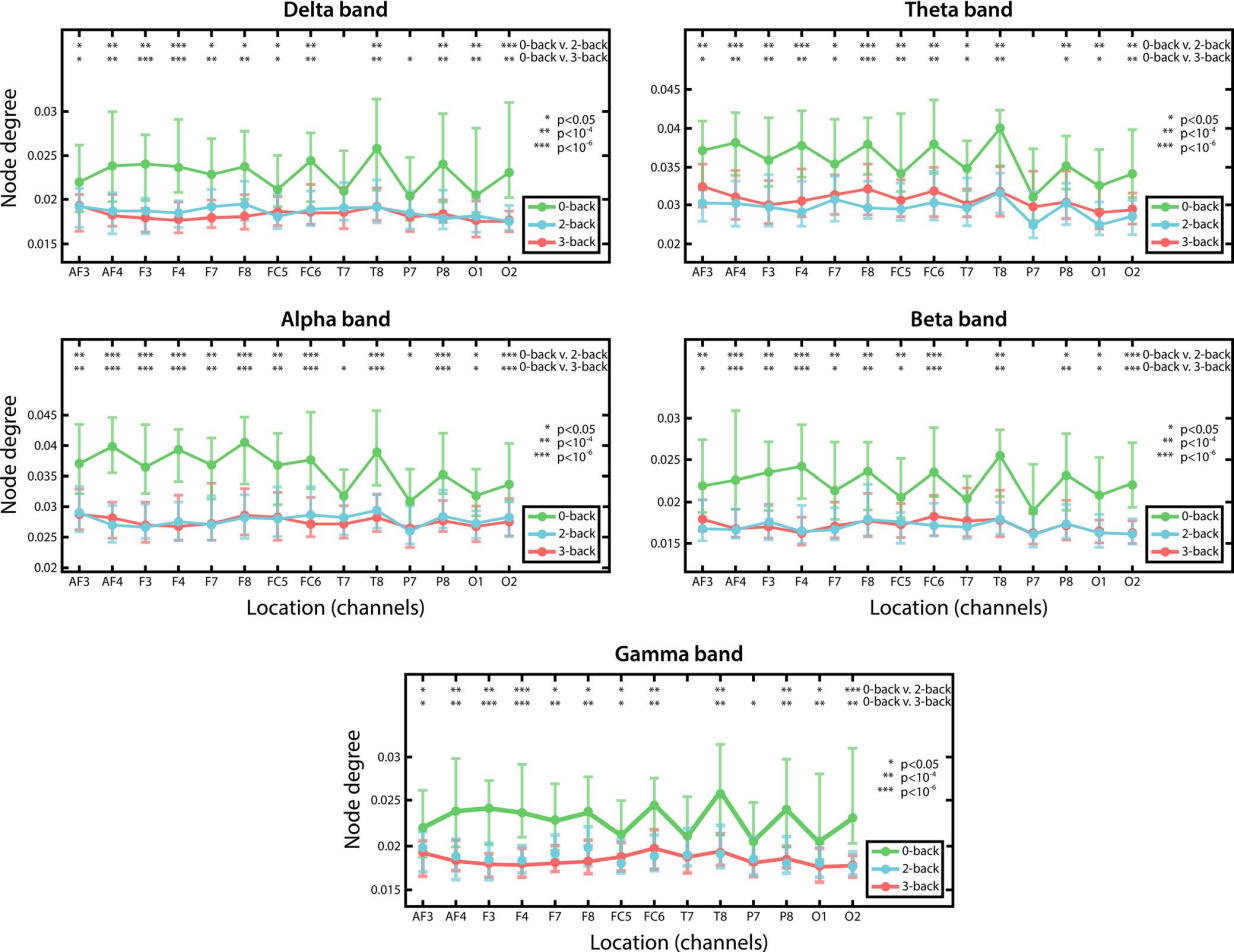
Weighted node degree in different n‐back conditions. Median*D_w_*values are plotted for all channels in all three task conditions, with results regarding 0‐back shown in black while those of 2‐ and 3‐back are shown in blue and red, respectively. The 0‐back condition could be characterized with higher weighted node degree than the 2‐ and 3‐back conditions over almost every cortical region. Significant pairwise differences between 0‐, 2‐ and 3‐back tasks are marked by *, ** and *** indicating*p* < .05,*p* < 10^–4^and*p* < 10^–6^following Bonferroni adjustment, respectively

Similarly to global network measures, values of *D_w_*, *C_w_* and *E_w_* were found strongly correlated, therefore we only present results regarding the weighted node degree, with those of the weighted clustering coefficient and local efficiency being highly comparable. In accordance with the results acquired for global network measures, *D_w_* was found significantly lower in the 2‐ and 3‐back conditions when compared to 0‐back, while no difference was found between the two difficulty levels (see Figure 5). These differences were found most pronounced over the frontal regions, while increased mental workload had more subtle or no effect on local connectivity over the temporal and parietal regions. Although Friedman tests indicated a significant effect of localization in all cases (*p* < .05), regional differences appeared less pronounced during the 2‐ and 3‐back tasks, corresponding to the decreased modularity found previously in these conditions.

### 3.4 Correlations with behavioral measures

**FIGURE 6:**
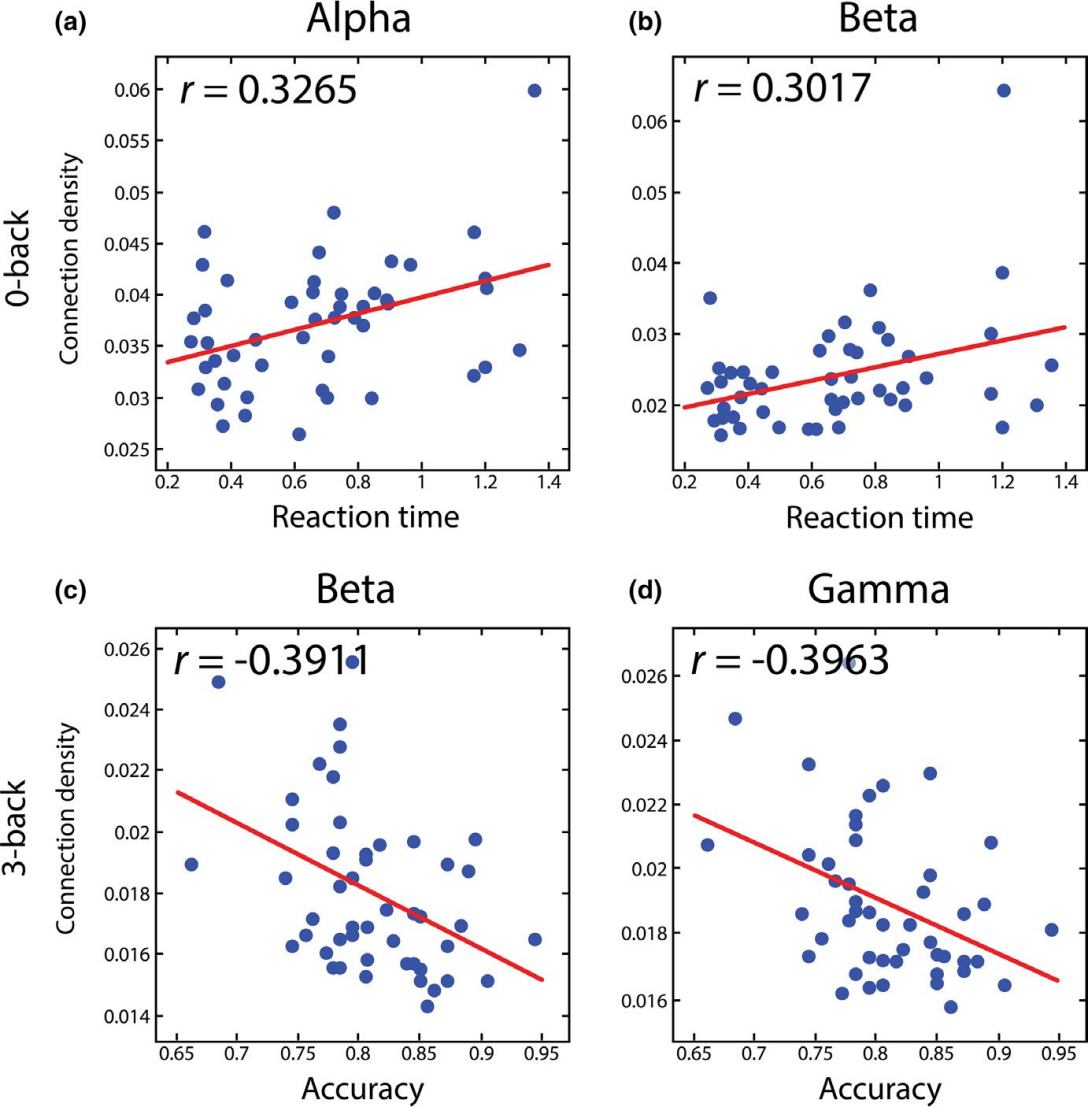
Correlations between behavioral measures and global functional connectivity. The positive relationship between reaction time and global connection density during 0‐back condition is shown for the alpha (a) and beta (b) bands. Conversely, accuracy showed an anticorrelated relationship with connection density during 3‐back in the beta (c) and gamma (d) bands. The value of the Spearman correlation coefficient*r*is indicated on each panel, as well as the trend acquired by least squares regression is plotted in red on all panels in order to illustrate the tendency of the relationship

We found significant positive correlations between RT and $\bar{D_{w}}$ in the alpha and beta bands (see Figure 6a,b), however only during the control condition (*r* = .3265, *p* = .0240 and *r* = .3017, *p* = .0376 for alpha and beta, respectively), while no relationship between RT and global FC was identified during the 2‐ and 3‐back tasks. On the other hand, accuracy was found negatively correlated to $\bar{D_{w}}$ in the beta and gamma bands (see Figure 6c,d) during 3‐back task performance (*r *= −.3911, *p* = .0060 and *r *= −.3963, *p* = .0053 for beta and gamma, respectively). Results acquired for $\bar{C_{w}}$ and $\bar{E_{w}}$ were identical, while no relationship was found between *Q* and any of the behavioral measures.

**FIGURE 7:**
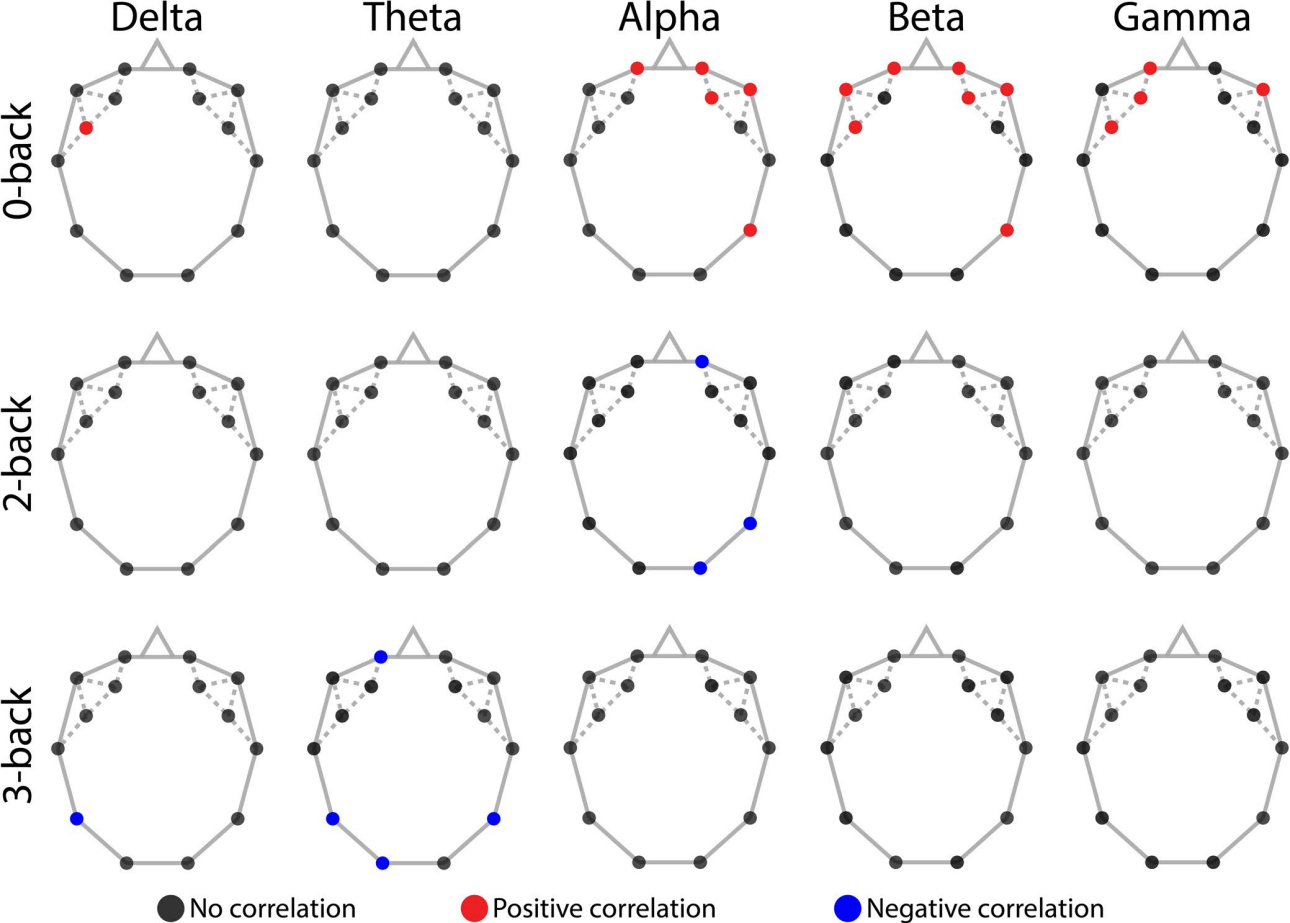
Correlations between reaction time and weighted node degree. Black dots indicate no correlation, while red and blue dots mark positive and negative correlation, respectively. The positively correlated nature of RT and*D_w_*in 0‐back (upper row) is well localized over the frontal regions in the alpha, beta and gamma bands. Negative correlations were also identified between RT and*D_w_*during 2‐ (middle row) and 3‐back (bottom row) in the alpha and theta bands, respectively, localized mainly over parietal and occipital regions. RT = reaction time;*D_w_* = weighted node degree

In order to explore the plausible relationship between FC and task performance in more detail, we also investigated the correlations between behavioral measures and local connectivity indices. This analysis indicated that the relationship between RT and $\bar{D_{w}}$ found previously could be attributed to the correlated nature of RT and *D_w_* during 0‐back, localized mostly over the frontal regions (see Figure 7). This pattern was present in the alpha, beta, and gamma bands. Interestingly, RT was found anticorrelated with *D_w_* during 2‐ and 3‐back over the occipital, frontal, and parietal regions in the theta and alpha bands (see Figure 7).

**FIGURE 8:**
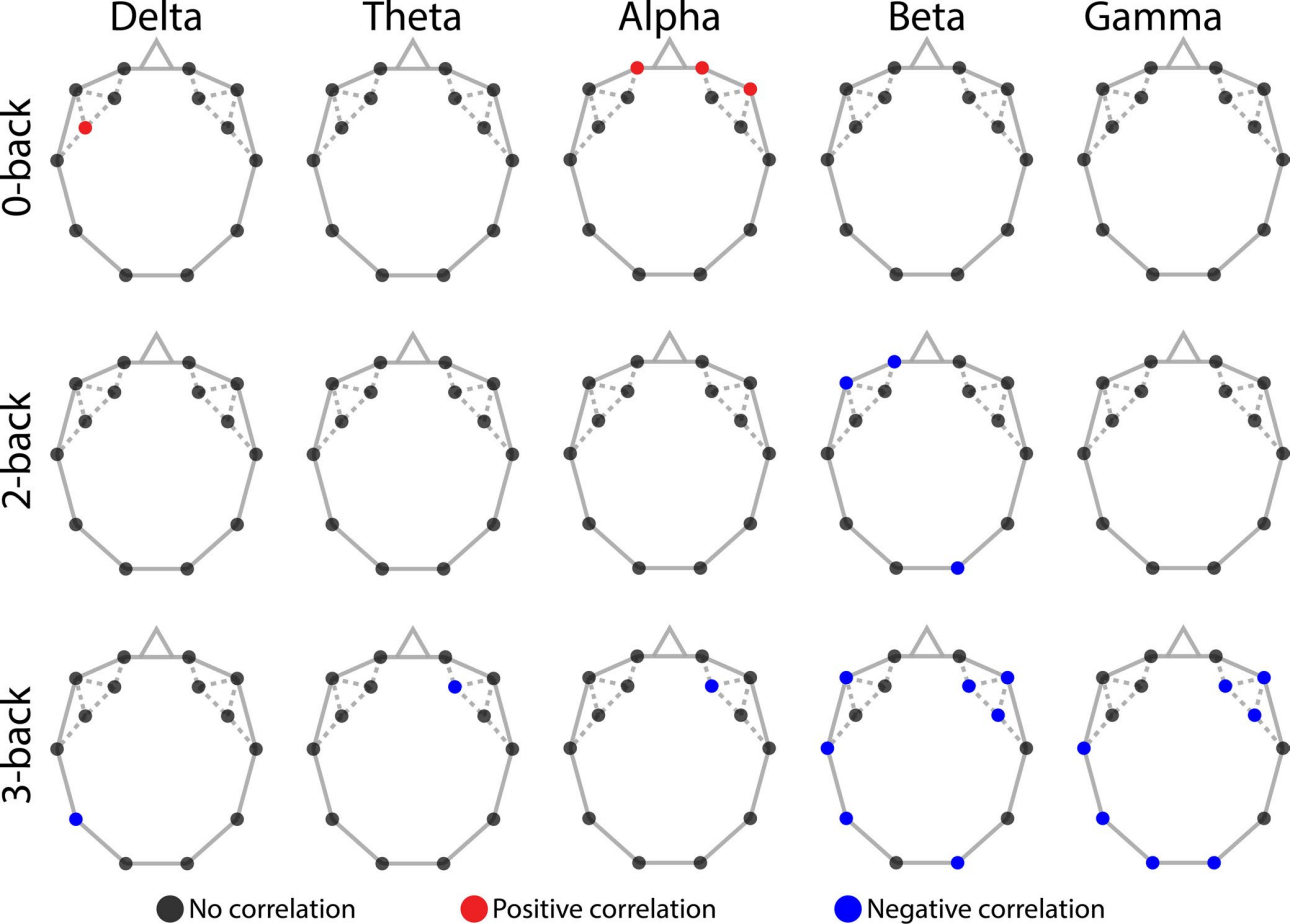
Correlations between accuracy and weighted node degree. Black dots indicate no correlation, while red and blue dots mark positive and negative correlation, respectively. The anticorrelated nature of ACC and*D_w_*in 3‐back (bottom row) is mainly localized over the occipital, parietal and right frontal regions in the beta and gamma bands. Positive correlations were also identified between ACC and*D_w_*during 0‐back (top row) in the alpha band, localized mainly over frontal regions. ACC = accuracy;*D_w_* = weighted node degree

Conversely, an anticorrelated relationship between ACC and *D_w_* was identified during 3‐back in the beta and gamma bands, restricted mostly to parietal, occipital and right frontal regions (see Figure 8). Additionally, ACC tended to show a negative correlation with *D_w_* during 0‐back over the frontal regions, as well as a positive correlation over F4 during 3‐back in the theta and alpha bands, while in this same task type showing negative correlation over P7 in the delta band (see Figure 8).

## 4 DISCUSSION

In this study, we report on a robust decrease of FC when implementing the *n*‐back WM paradigm in all the five conventional frequency bands when compared to the control condition. Local connectivity analysis indicated that this effect could be attributed mostly to a decrease in FC over the prefrontal and frontal regions. Conversely, no effect of task difficulty on FC could be identified. Network topological measures such as density, clustering coefficient, and global efficiency yielded highly consistent results, indicating that at this resolution they generally captured the same information. On the other hand, the obtained modularity index indicated that while in the control (0‐back) condition a frontal and an occipitotemporal cluster of nodes could be distinguished, this modular structure practically got dissociated during the 2‐ and 3‐back task conditions. The RT of the subjects was found positively correlated to FC in the alpha and beta bands, however only during the control state. In contrast, subject accuracy was found negatively correlated with FC in the beta and gamma bands, although only in the most challenging setting (3‐back).

Our results are in line with earlier reports on decreased connectivity during the *n*‐back WM paradigm (Dai et al., 2017; Ginestet & Simmons, 2011; Hou et al., 2018). However, in contrast to Dai et al. (2017), we found a stimulation‐related decrease in E and C in both the theta and alpha bands and in the beta band, respectively, while Hou et al. (2018) found an increase in the same measures. These seemingly contradictory results may be attributed to differences in the analysis pipeline, which can affect results significantly even when obtained from the same dataset (Jalili, 2016; Lindquist, 2020). In the first study (Dai et al., 2017), FC was assessed as the Pearson correlation—a linear measure of statistical interdependence—of band‐limited power time series following source reconstruction. Instead, our approach was carried out in the electrode space using PLI as the FC estimator, a measure of nonlinear phase coupling; therefore, a direct comparison would be difficult. Still, in order to explore the plausible effect of the FC estimator, we re‐analyzed the current dataset using a different, linear measure of interdependence, spectral cross‐coherence (Srinivasan et al., 2007). We found nearly identical results at both global and local levels as well as regarding correlations with behavioral variables, rendering it highly improbable that the choice of FC estimator added a significant bias to our findings.

On the other hand, Hou et al. (2018) estimated FC in the electrode space using PLI, a pipeline strongly similar to ours. However, in that study, the authors only used epochs of 2.5 s taken from the beginning of each stimulation block, in addition to estimating the graph theoretical measures first and averaged them over various difficulty settings subsequently. As shown previously, that FC characteristics can vary significantly in different phases of various WM tasks (Ren et al., 2017; Toppi et al., 2018; Zhang et al., 2019); therefore, it is plausible that the functional organization in the brain at the beginning of each stimulation block is different from later periods. Also, in our analytical pipeline, the obtained synchronization matrices were first averaged over the stimulation blocks of corresponding difficulty levels, and network measures were only then calculated from the average connectivity matrices. This procedure was shown to yield statistically more robust and reproducible connectivity estimates (Hardmeier et al., 2014). Therefore, by utilizing this technique a better assessment of the characteristic FC pattern at each difficulty level could be achieved, similarly as when increasing the signal‐to‐noise ratio through block‐averaging (Buckner et al., 1996). Nevertheless, to further test this hypothesis, we re‐evaluated our results by calculating network measures from the estimated connectivity matrices first and then averaging network measure values for corresponding task difficulties. This analysis produced nearly identical results for *D_w_*, *C_w_* and *E_w_*. In contrast, although the same decreasing tendency of *Q* with increasing the difficulty was apparent, the differences were found nonsignificant in all scenarios. This suggests that averaging synchronization matrices first to obtain the average connectivity patterns of each state indeed yields a more robust characterization of network topology. Also, we found a strong correspondence between *D_w_*, *C_w_* and *E_w_* in all frequency bands. This may indeed suggest that these three network measures basically capture the same information on small networks such as those analyzed in this study (Hardmeier et al., 2014; van Wijk et al., 2010), where network size prevents the full and robust manifestation of network characteristics such as segregation or integration (Rubinov & Sporns, 2010).

Contrary to most studies, Fishburn et al. (2014) reported increased FC during *n*‐back using fNIRS for monitoring neural activity. However, when comparing these results, the very different mechanisms involved in measuring evoked cognitive responses by fNIRS and EEG should be taken into consideration. Specifically, fNIRS is able to measure changes in regional blood volume that is elicited by an increase in local neuronal activity, thus it provides an indirect manner to investigate neural dynamics (Irani et al., 2007; Keles et al., 2016). Due to the low‐pass filtering effect of the neurovascular coupling (Huneau et al., 2015), the frequency range of neural activity‐related components captured by fNIRS is approximately two orders of magnitude lower (usually between 0.01–0.1 Hz) than of those directly monitored by EEG (~1 Hz and higher). Moreover, it has been shown that neural fluctuations express markedly different dynamic properties in these frequency ranges when investigated by EEG and fNIRS (Nagy et al., 2017). Hence, it can be hypothesized that these contradictory results may emerge due to the characteristics of the imaging modalities. This is also in line with our previous study reporting on increased FC during a pattern recognition‐based WM task when assessed by fNIRS imaging (Racz et al., 2017).

Nevertheless, our findings yet remain contradictory to some extent when compared to those of the literature, indicating that further research is called for. Future studies may focus on the influence of various preprocessing and analysis pipelines during the n‐back paradigm. A relevant future direction may be the extension of such pipelines to the framework of dynamic FC (Preti et al., 2017), which would allow for monitoring how FC changes in time throughout a longer stimulation block. A further aspect that could be taken into consideration is subject fatigue or stress, which may have an influence not only on performance measures but also on how the brain responds to increased mental challenge (Dimitrakopoulos et al., 2018). Although our analyses revealed a marked decrease in FC even when only limited number of cortical regions were monitored, the effects of electrode density may be better assessed in a high‐density setup utilizing multiple analysis pipelines, each using data from various subsets of all electrodes. Simultaneous monitoring of EEG and NIRS (Wallois et al., 2012) during n‐back may help resolve contradictory findings when analyzing neural signals of different modalities. Finally, alterations in physiological parameters when adapting to increased mental workload could also be considered in a broader context, focusing not only on changes occurring in the brain but on the level of the entire organism. It has been shown that cognitive stimulation may evoke involuntary responses in the cardiorespiratory (Debreczeni et al., 2009; Szirmai et al., 2005) or autonomic nervous systems (Hjemdahl et al., 1989), that may also affect indices derived from neural signals. The recently introduced concept of network physiology (Bartsch et al., 2015; Bashan et al., 2012; Ivanov & Bartsch, 2014) provides a framework able to account for such interactions, namely how different organ systems dynamically interact during physiological functioning. Indeed, it has been shown that characteristic brain connectivity patterns across multiple frequency ranges could be associated with various global physiological states, such as sleep stages (Lin et al., 2020). Clearly, cognitive studies carried out in a network physiological framework—for example, the work of Zanetti et al. (2019)—could not only reveal global physiological states associated with mental processes, but may also help in resolving some of the controversies regarding functional brain connectivity during increased mental workload.

An interesting finding of this study is the presence of two distinct modules—frontal and occipitotemporal—in the control condition that dissociates when cognitive demand increases. This observation is in contrast with the hypothesis that since WM tasks require the involvement of multiple functions such as information encoding and retrieval, short‐term memory and increased attention (Roux & Uhlhaas, 2014), an increase in FC during a WM task would be reasonable as being consistent with enhanced cooperation between various brain regions. On the contrary, connectivity strength is more often found decreased while performing an *n*‐back task (Cohen & D'Esposito, 2016; Ginestet & Simmons, 2011). An explanation for this phenomenon is offered by the fact that the brain is highly active and densely connected even in the resting state, that is, in the absence of any external stimuli (Damoiseaux et al., 2006; Raichle et al., 2001). Namely, the set of brain regions most consistently found functionally coupled in the resting state is often termed the default mode network (DMN), consisting mainly of the medial PFC, the posterior cingular cortex, the precuneus, and the angular gyrus (Buckner et al., 2008). A fundamental characteristic of the DMN is that it is practically dissolved (i.e., its FC decreases rapidly) in the presence of external stimuli (Fox et al., 2005). This notion is partially in support of our findings, especially considering that modules identified in the control condition correspond well to regions of the DMN. However, an exact correspondence cannot be established since the control condition itself (0‐back) should not be considered as a purely resting‐state condition. Maintaining such a dense network of functional connections in the resting state requires high metabolic expenditure. Therefore, under increased mental workload when computational demands are increased even further, connections that are not vital for performing the actual task are likely being “pruned” in order to allow for the recruitment of new connections specifically required to solve the task (Cohen & D'Esposito, 2016; Fox et al., 2005). Additionally, it has been shown recently that FC between various subnetworks (A and B) of the frontoparietal network (FPN) and the DMN and dorsal attention network (DAN) may influence cognitive performance in a WM task (Murphy et al., 2020). Specifically, activity of FPN subnetwork B was found inversely correlated with FC between the DMN and FPN, while positively correlated with FC between DAN and FPN (Murphy et al., 2020). Consequently, the observed decrease of FC in our study—that may reflect decreased DMN activity—may also indicate an increased activity of FPN subnetwork B and DAN, both systems crucial in adapting to increased WM demands (Dixon et al., 2017; Palva et al., 2010). This may also be partially supported by the fact that we found a negative correlation between FC and subject accuracy, as an anticorrelated relationship between activities of the DMN and the FPN is associated with increased cognitive performance (Anticevic et al., 2010). Furthermore, although modularity decreased during task performance, it expressed no significant correlation with behavioral measures. In a recent study it has been shown, that even though modular structure of brain networks is critical for cognitive performance, global modularity (as captured in *Q*) only showed weak correlations with task performance in a 2‐back WM task (Bertolero et al., 2018). Instead, connectivity of specific regions acting as local or connector hubs—and thus attuning global modularity of the network—could be effectively utilized for accurately predicting cognitive performance (Bertolero et al., 2018). These results clearly indicate the key role of network modularity in cognitive performance, while the lack of relationship between behavioral measures and Q in our study was most likely due to the small network size preventing the manifestation of more elaborate network topological features.

Theta activity has been proposed to be responsible for controlling and integrating various WM functions (Sauseng et al., 2010). This hypothesis is supported by multiple studies reporting on increased theta‐band activity and enhanced theta‐connectivity during increased mental workload (Dai et al., 2017; Langer et al., 2013; Sauseng et al., 2005). While we found a significant decrease of theta‐band FC in the task when compared to the control condition, a slight (nonsignificant) tendency of higher FC also appeared in the 3‐back when compared to the 2‐back condition (see Figure 4). The average connectivity matrices showed that this could be attributed mainly to stronger connections within frontal and prefrontal regions (see Figure 4), consistent with previous reports on increased theta activity over the same regions. In contrast, alpha‐band activity during WM tasks is considered responsible for the suppression of neural activities unrelated to task performance and thus attention control (Klimesch et al., 2007), which is in line with our findings along with those of other studies reporting on decreased alpha‐band connectivity during WM tasks (Dai et al., 2017; Hou et al., 2018). Higher frequency (beta‐ and gamma‐band) neural activity and connectivity were found to be related to cognitive performance, high executive demand, increased attentiveness, and maintenance of WM information (Guevara et al., 2018; Makeig & Jung, 1996; Roux & Uhlhaas, 2014). These previous findings suggest that neural activity at various frequency ranges plays roles in fundamentally distinct aspects of cognitive functioning. Surprisingly, our results regarding global and local FC revealed a generally similar pattern of changes in all investigated frequency bands during different *n*‐back conditions (although the actual values of FC measures varied substantially between the various ranges, see Figure 3). Nevertheless, the former notion is supported by the fact that FC indices showed relevant correlations with behavioral measures only in the alpha, beta, and gamma bands. Specifically, RT was positively correlated with alpha‐ and beta‐band FC, however only during the control condition, with the inference being that stronger alpha and beta synchronization predisposes longer RTs in the absence of cognitive demand. However, this relationship gets dissolved with decreasing alpha and beta synchronization in the presence of more involved WM functions. Conversely, there was an inverse relationship between cognitive performance (as measured in the subject accuracy) and FC in the beta‐ and gamma bands, however, this correspondence only became prominent at the hardest difficulty level, 3‐back. This implies that as cognitive demand increases, increased large‐scale connectivity of high‐frequency neural activity would lead to poorer performance.

Finally, we must address some of the limitations and future perspectives of this study. EEG recordings were carried out by using a low‐density setup with moderate spatial (14 channels) and temporal (128 Hz) resolution. On the one hand, this poses an obvious limitation, that underlies the fact that network measures *D_w_*, *C_w_* and *E_w_*—that otherwise characterize vastly different aspects of network topology—yielded highly similar results. On the other hand, this highlights the robustness of our results, which indicated a very powerful effect of increased mental workload (*p* < 10^–4^ even following Bonferroni adjustment) on FC in the vast majority of cases. Also, our experimental paradigm did not contain a purely resting‐state condition. Therefore, an important future direction is to expand our analyses on how large‐scale connectivity patterns are reorganized when transitioning from resting state to control (0‐back) and increased mental workload (2‐ and 3‐back) states. Another important aspect to consider is test‐retest reliability, which in the case of FC and graph theoretical studies (such as this study) so far appears to be highly consistent (Hardmeier et al., 2014; Vecchio et al., 2020). Additionally, FC has been shown previously to vary not only in response to stimuli (Sakoglu et al., 2010) but also to fluctuate even in the resting state (Chang & Glover, 2010; Hutchison et al., 2013). Furthermore, dynamic graph theoretical analysis (Dimitriadis et al., 2010) has been successfully applied to reveal nontrivial features of dynamic FC such as its (multi)fractality (Racz, Mukli, et al., 2018; Racz, Stylianou, et al., 2018) or (spatially varying) information content (Racz et al., 2019, 2020). Since fractal aspects of brain dynamics have been shown to correlate with cognitive stimulation (He, 2011; He et al., 2010), the question of how these novel features of dynamic FC could be utilized in characterizing the functional organization of the brain during various WM paradigms appears important to pursue.

## 5 CONCLUSIONS

In this study, we found that large‐scale FC of the brain decreased with increasing mental workload, however, this was found independent from the difficulty of the applied WM task. This decrease in FC could be attributed to the dissolution of two—a frontal and an occipitotemporal—functional modules that were only present in the control condition, while during increased mental workload the functional network of the brain expressed a more homogenous topology. This response may reflect the suppression of functional connections irrelevant to task solving. Behavioral variables showed correlations only with high‐frequency FC. In that, RT was positively correlated with alpha‐ and beta‐band FC only in the control state, while accuracy was negatively correlated with beta‐ and gamma‐band FC only in the hardest difficulty setting. These results indicate that an impaired downregulation of FC may result in poorer performance.

## CONFLICT OF INTEREST

The authors declare no conflict of interest.

## AUTHOR CONTRIBUTIONS

ZK contributed to data collection, data analysis, interpretation of the results and manuscript preparation. OS contributed to data collection and preprocessing. PM contributed to study design and statistical analyses. AE provided conceptual guidance and critically supervised the study. FSR contributed to conceptualization, data analysis, interpretation of the results and manuscript development. All authors provided revisions and gave their approval on the final version of the manuscript before submission.

### Peer Review

The peer review history for this article is available at https://publons.com/publon/10.1002/brb3.1932.

## Data Availability

The data that support the findings of this study are available from the corresponding authors upon reasonable request.
